# Immune response and drug therapy based on ac4C-modified gene in pancreatic cancer typing

**DOI:** 10.3389/fimmu.2023.1133166

**Published:** 2023-03-06

**Authors:** Dong Xu, Kaige Huang, Yang Chen, Fei Yang, Cunbing Xia, Hongbao Yang

**Affiliations:** ^1^Department of General Surgery, Gaochun People’s Hospital, Nanjing, Jiangsu, China; ^2^Department of General Surgery, Affiliated Hospital of Nanjing University of Chinese Medicine, Jiangsu Province Hospital of Chinese Medicine, Nanjing, Jiangsu, China; ^3^Department of Pharmacy, Nanjing University of Chinese Medicine, Nanjing, Jiangsu, China; ^4^Center for New Drug Safety Evaluation and Research, Institute of Pharmaceutical Science, China Pharmaceutical University, Nanjing, China

**Keywords:** pancreatic ductal adenocarcinoma, NAT10, ac4C, bioinformatics, tumor environment

## Abstract

N-4 cytidine acetylation (ac4C) is an epitranscriptome modification catalyzed by N-acetyltransferase 10 (NAT10) and is essential for cellular mRNA stability, rRNA biosynthesis, cell proliferation, and epithelial-mesenchymal transition (EMT). Numerous studies have confirmed the inextricable link between NAT10 and the clinical characteristics of malignancies. It is unclear, however, how NAT10 might affect pancreatic ductal adenocarcinoma. We downloaded pancreatic ductal adenocarcinoma patients from the TCGA database. We obtained the corresponding clinical data for data analysis, model construction, differential gene expression analysis, and the GEO database for external validation. We screened the published papers for NAT10-mediated ac4C modifications in 2156 genes. We confirmed that the expression levels and genomic mutation rates of NAT10 differed significantly between cancer and normal tissues. Additionally, we constructed a NAT10 prognostic model and examined immune infiltration and altered biological pathways across the models. The NAT10 isoforms identified in this study can effectively predict clinical outcomes in pancreatic ductal adenocarcinoma. Furthermore, our study showed that elevated levels of NAT10 expression correlated with gemcitabine resistance, that aberrant NAT10 expression may promote the angiogenic capacity of pancreatic ductal adenocarcinoma through activation of the TGF-β pathway, which in turn promotes distal metastasis of pancreatic ductal adenocarcinoma, and that NAT10 knockdown significantly inhibited the migration and clonogenic capacity of pancreatic ductal adenocarcinoma cells. In conclusion, we proposed a predictive model based on NAT10 expression levels, a non-invasive predictive approach for genomic profiling, which showed satisfactory and effective performance in predicting patients’ survival outcomes and treatment response. Medicine and electronics will be combined in more interdisciplinary areas in the future.

## 1 Introduction

Pancreatic ductal adenocarcinoma (PDAC) is the 12th most common malignancy and the 7th leading cause of cancer death worldwide with a 5-year survival rate of only 10% (1–4). Pancreatic ductal adenocarcinoma has more than doubled in the last 25 years and now ranks among the top 10 cancer killers in more than 130 countries (5). According to recent data from the American Cancer Society, there were approximately 60,430 new cases of pancreatic ductal adenocarcinoma and approximately 48,220 deaths in 2021, which are expected to be the second leading cause of cancer deaths in the United States in the next 20 – 30 years. As the third leading cause of cancer-related deaths in the European Union, pancreatic ductal adenocarcinoma is expected to surpass breast cancer (6). We also face multiple challenges in treatment, with surgical resection, while potentially curative, often requiring combined therapeutic interventions (7). In resectable or potentially resectable pancreatic ductal adenocarcinoma, preoperative radiotherapy does not provide a significant overall survival benefit (8). Although combination chemotherapy may improve overall survival, its adverse effects can severely affect patients’ quality of life, thus limiting its clinical use to a certain extent (9). A recent study has shown that targeting the components of the tumor mesenchyme that cause connective tissue proliferation can have some therapeutic effects (10), for example, polymeric nanoparticles, which can be intelligently designed with carriers that significantly reduce the difficulty of crossing the stromal barrier and thus improve drug delivery (11). As standard treatments such as conventional chemotherapy, radiotherapy, surgery, and targeted therapies do not provide long-term remission of pancreatic ductal adenocarcinoma, there is an urgent need to develop new therapeutic strategies to improve the prognosis of pancreatic ductal adenocarcinoma patients.

NAT10 belongs to the GCN5-related N-acetyltransfer (GNAT) family of acetyltransferases. Previous research has shown that NAT10 catalyzes the acetylation of histones, mRNA, and other substrates and is involved in a variety of cellular processes such as ribosome production, DNA damage repair, cytoplasmic division, and mRNA translation regulation (12). NAT10, as an epigenetic regulator, may aid in the treatment of cancer and osteoporosis (13). It is unclear, however, what role NAT10 plays in PDAC.

In this study, we aimed to reveal the correlation between NAT10 expression levels and the clinical characteristics of pancreatic ductal adenocarcinoma patients. We collected genomic data from TCGA, GTEx and GEO databases of pancreatic ductal adenocarcinoma and then constructed NAT10 subgroup phenotypes by unsupervised clustering of NAT10-mediated gene expression levels of ac4C modifications. The expression levels of NAT10 were confirmed to be associated with tumor tissue immune infiltration, clinical outcome, drug resistance, tumor cell migration and clonogenic ability. In summary, our findings reveal a crucial role for NAT10 in pancreatic ductal adenocarcinoma, and we propose a convenient method to help diagnose and predict survival outcomes of pancreatic ductal adenocarcinoma patients.

## 2 Materials and methods

### 2.1 Multi-omics cohort of pancreatic ductal adenocarcinoma from TCGA

The cohort contains molecular profiles from the Cancer Genome Atlas (TCGA) PDAC dataset containing transcriptome data from 185 patient samples (14). We then retrieved for TCGA cohort with multi-omics profiles, including transcriptome expression, somatic mutations, copy number alterations (CNAs), clinicopathological features and clinical outcomes. From UCSC Xena (http://xena.ucsc.edu/), we downloaded gene expression data for 165 primary PDAC cases. Transcripts per kilobase million (TPM) values were then converted from FPKM to improve comparability between samples and show greater similarity to microarray results (15). GENCODE22 mapping from UCSC Xena was used to convert Ensembl IDs for transcriptomes into gene symbols. Furthermore, a copy number segment file was obtained from FireBrowse (http://firebrowse.org/). By using cBioPortal, we collected data on somatic mutations, clinicopathological features, progression-free survival (PFS), and overall survival (OS). (https://www.cbioportal.org/).

### 2.2 External validation cohorts of PDAC

To test the reproducibility of our identified molecular subtypes, we collected a total of five external independent cohorts, including PACA_AU (n = 269) which was downloaded from International Cancer Genome Consortium (ICGC) (16), E-MTAB-6134 containing 309 consecutive patients with PDAC from a multi-center consortium (17), and three cohorts from the Gene Expression Omnibus (GEO) database; the GEO datasets included GSE71729 (n = 125) (18), GSE62452 (n = 66) (19), and GSE57495 (n = 63) (20). From the corresponding archives, we retrieved clinical outcomes only for PDAC patients and excluded those with neuroendocrine or acinar cell carcinomas. When multiple probe IDs were associated with a gene symbol in three microarray datasets from the GEO database, the median value was taken into account. The three independent GEO datasets were combined considering the relatively small sample size. Potential cross-dataset batch effects were eliminated by the R package sva under an empirical Bayesian framework and batch effects were further investigated using principal component analysis (21).

### 2.3 A list of genes modified by N-Acetyltransferase 10

To many molecular components, NAT10 was known as an acetyltransferase. A recent study discovered NAT10’s ability to acetylate mRNA, providing new insight into the epitranscriptome. The literature yielded a list of 2,156 genes that are altered by NAT10-mediated ac4C (22).

### 2.4 Tumor microenvironment composition analysis

The R package MCPcounter was used to assess TME composition in PDAC patients (23). An analysis of transcriptomic markers determines the scores of a given cell population, which are transcriptomic features that are strongly, specifically, and consistently expressed. Based on these scores, large cohorts and inter-sample comparisons can be made between tumor populations (24). We calculated infiltrating immune/stromal cells and tumor purity in tumor tissue using the R package “estimate” (25). In practice, it is frequently difficult to obtain cancer samples with sufficient tumor purity, particularly for PDAC. In this context, we kept those tumor samples with tumor purity of greater than 70% to ensure sufficient tumor cells and microenvironment cells for analytic purposes.

### 2.5 An analysis of gene signatures based on functional orientation

The functional orientation of the TME was determined using literature signatures (26). Immune suppression (CXCL12, TGFB1, TGFB3, and LGALS1), T cell activation (CXCL9, CXCL10, CXCL16, IFNG, and IL15), T cell survival (CD70 and CD27), regulatory T cells (FOXP3 and TNFRSF18), major histocompatibility complex class I (HLA-A, HLA-B, HLA-C, HLA-E, HLA-F, HLA (CXCL13). The geometric mean signature expression was used to compute scores for each signature. Additionally, DNA methylation scores were calculated using protocols described in the literature for tumor-infiltrating lymphocytes (MeTILs) (27).

### 2.6 Intracohort immune classifications

Unsupervised hierarchical clustering of samples in the discovery TCGA cohort was performed using Euclidean distance and Ward linkage criteria based on polygenic abundance scores from MCPcounter included cohorts (28). Based on the dendrograms and the relatively small sample size, three clusters were empirically determined.

### 2.7 Calculation of replication stress enrichment

Using single-sample gene set enrichment analysis (ssGSEA), we identified 21 replication stress signatures from the literature (29). Hierarchical clustering identified two replication stress subtypes.

### 2.8 Analysis of differential expression and gene set enrichment

A differential expression analysis was performed using the R package “limma” (30). We created a pre-ranked gene list based on log2FoldChange values derived from differential expression analysis for gene set enrichment analysis (GSEA). The Hallmark pathway was used to determine functional enrichment using the R package clusterProfiler (31, 32).

### 2.9 Cancer subtype characterization and visualization

We performed comprehensive characterization on subtypes identified from multi-omics aspect using the R package MOVICS (33), including prognosis, mutational landscape, chromosomal stability and clinicopathological features. Specifically, for mutational landscape, significantly, differentially, and frequently mutated genes among subtypes were identified (mutational rate > 3% with *P* < 0.05). The individual fraction of copy number-altered genome (FGA) was calculated to represent chromosomal instability based on copy number segment data using the threshold of 0.2 (33). In addition, we detected and localized recurrent focal somatic CNAs by GISTIC2.0 through GenePattern with criteria by default as the following: threshold for amplification or deletion: 0.1, *q*-value: 0.25 with 75% confidence level (https://www.genepattern.org/) (34).

### 2.10 Nearest template prediction

In order to classify genes based on gene expression, nearest template prediction was used (NTP). This prediction algorithm provides a convenient model-free approach that uses only feature gene lists and test datasets for single-sample class prediction and is flexible and beneficial in external cohort applications (35, 36).

### 2.11 Therapeutic response analyses

We used the R package to analyze chemical compounds. Drug sensitivity for PDAC was predicted for the TCGA cohort using the R ‘pRRophetic’ package based on GDSC drug sensitivity and phenotypic data (https://www.cancerrxgene.org/). In the R package, ridge regression was used to estimate the AUCs for each sample treated with a particular chemotherapeutic agent (37).

For immunotherapy especially to immune checkpoint inhibitors, we collected a transcriptome profile and clinical response of 47 patients with melanoma who treated with either anti-PD1 or anti-CTLA4 immunotherapies (38). A comparison of transcriptome profiles enabled us to predict the clinical response to immune checkpoint inhibitors using subclass mapping (39).

### 2.12 Differential expression of NAT10 correlates with prognosis

A total of 179 PDAC and 332 standard pancreatic tissue specimens from the TCGA and GTEx databases, as well as OS, PFS, DFS, and Disease Special Survival (DSS), were selected to assess the correlation between differential expression of NAT10 and prognosis. The human protein atlas: This database, also known as the human protein expression atlas, is a study of the immunohistochemical staining status of proteins in normal human tissue, cancer tissue and cancer cell lines, allowing for differential protein expression analysis in tumor tissues, and for Gene and tumor survival analysis. In this study, immunohistochemical analysis of NAT10 protein in PDAC tissues was performed using The human protein atlas database.

### 2.13 Cell culture

we analyzed the expression of NAT10 in tumor and normal tissues, human Pancreatic ductal adenocarcinoma cell lines (Capan-1, CFPAC, PANC-1, MIAPaCa-2 and BXPC-3) and pancreatic duct epithelial tissue cell line (HPDE6-C7) were obtained from BeNa Culture Collection (Beijing, China). Capan-1 was maintained in Iscove’s Modified Dulbecco’s Medium (IMEM) supplemented with 20% Fetal Bovine Serum (FBS) and HPDE6-C7 was maintained in Dulbecco’s Modified Eagle Medium (DMEM) supplemented with 10% FBS, at 37°C in 5% CO_2_.

### 2.14 Immunohistochemistry staining

All samples used in this study were approved by the Ethics Committee of Shanghai Outdo Biotechnology Co., Ltd (No. HPanA120Su02). The operation time was from January 2009 to August 2013, and the follow-up time was from November 2014, with a follow-up period of 1.2 to 5.8 years. A total of 66 cases of cancerous tissue and 54 cases of paracancerous tissue were obtained for immunohistochemistry. Pancreatic ductal adenocarcinoma tissues collected were fixed with 4% paraformaldehyde, dehydrated, embedded into paraffin blocks and sliced into 4μm. The sections were hydrated after dewaxing and incubated with 3% H_2_O_2_. After antigen repair and being blocked, slides were blocked and incubated overnight at 4°C with rabbit antibody specific for NAT10 (1:100) (Abcam, USA). For 20 minutes, the sections were incubated with a streptavidin-biotin-peroxidase complex, followed by 30 minutes with the secondary antibody. We stained the slide with 3, 3-diaminobenzidine (DAB) substrate kit for a peroxidase reaction and counterstained with hematoxylin. The slides were analyzed under an optical microscope with a digital camera.

### 2.15 Detection of mRNA expression levels by RT-qPCR

The total RNA was extracted using the Trizol kit according to the instructions, and its concentration and purity were checked using the Nano-300 microspectrophotometer. Cells were used for subsequent experiments after qRT-PCR was used to measure the expression levels of target RELATIVE NAT10 mRNA. The used specific primer sequences were as follows: NAT10-forward primer (GAGACAGACCCCGAATGACC), reverse primer (GGAGAGCAAGGCTAGGAACC) and GAPDH-forward primer (GGAGCGAGATCCCTCCAAAAT), reverse primer (GGCTGTTGTCATACTTCTCATGG).

### 2.16 Western blot analysis

Cells were collected and lysed on ice with a mixture of RIPA lysate(Thermo Fisher, USA) and PMSF(Beyotime, ST506, China), total protein was extracted from each group of cells, and the BCA kit(Thermo Fisher, USA) was used to determine the protein content of each group. 40μg of the sampled proteins were subjected to SDS-PAGE, 250 mA constant flow membrane transfer (0.22 μm PVDF), and closed with TBST blocking solution (containing 50 g/L skimmed milk powder) for 2 h. Then the following primary antibodies were incubated overnight at 4°C: rabbit monoclonal anti-NAT10 (1 : 1000, ab194297, Abcam), rabbit monoclonal anti-β-actin (1 : 1000, 4970, Cell Signaling Technology), the next day the cells were rinsed with TBST and the secondary antibody was added for 2 h at room temperature. Using an ECL kit(Thermo Fisher, USA), the expression of the target bands was detected. Use the ImageJ software (version 1.50b; National Institutes of Health) for grayscale analysis. Each experiment was repeated three times to minimize experimental error.

### 2.17 Interference with NAT10 by transient transfection

Small interfering RNA (siRNA) was transfected into Capan-1 cells by Lipofectamine 3000. Cells were collected, and NAT10 protein expression levels were determined using Western blot. siRNAs were obtained from Shanghai Genepharma. Sequences of siRNA were as following: siRNA1 NAT10:sense 5′-GGCAGACUAUUCAGUAUAUTT-3′, anti-sense 5′-AUAUACUGAAUAGUCUGCCTT-3′; siRNA2 NAT10:sense 5′-GGCCAAAGCUGUCUUGAAATT-3′, anti-sense 5′-UUUCAAGACAGCUUUGGCCTT-3′; Negative control: sense 5′-UUCUCCGAACGUGUCACGUTT-3′, anti-sense 5′-ACGUGACACGUUCGGAAATT-3′.

### 2.18 Transwell assay

Cells from the above groups were digested 48 h after transfection, collected in serum-free DMEM medium and counted, and diluted to 2.5×10^4^ cells/100 μL. 700 μL of complete medium (DMEM with 100 mL/LFBS) was added to the lower chamber, 8 μm diameter TranswellTM cells were placed in a 24-well plate, and 200 μL of the serum-free medium was added to the upper chamber. After incubation for 24h, the chambers were removed and fixed in 40g/L paraformaldehyde for 30min; 1g/L crystal violet was used for staining for 30min.

### 2.19 Colony formation assay

Using a 6 cm plate with 1000 cells inoculated and incubated at 37°C, clone formation was assessed by number of colonies formed. According to the characteristics of each cell line, the medium was changed regularly after 10-15 days. We washed the adherent cells twice in PBS, fixed them with 4% paraformaldehyde, and stained them with 0.1% crystal violet (Sigma-Aldrich, NY, USA). The total number of colonies (50 cells per colony) was counted using Image J software.

### 2.20 Statistical analyses

R was used to conduct statistical analyses (v4.0.2). P values or adjusted P values less than 0.05 were considered significant for all statistical comparisons. Mann-Whitney U tests were used to compare two conditions, and Kruskal-Wallis tests were used to compare three conditions. The independence of categorical variables was determined using Fisher’s exact tests. To analyze Kaplan-Meier survival curves and log-rank test calculations, two-sided log-rank P values were calculated using the R package survminer. Each experiment was repeated three times to minimize experimental error. Data are shown as mean ± standard error of the mean (SE). The 2^-ΔΔ Ct^ method was used to analyze the results of real-time PCR in all of the experiments.

## 3 Results

### 3.1 The identification of prognostic subtypes in PDAC based on NAT10-relevant genes

**Figure 1:**
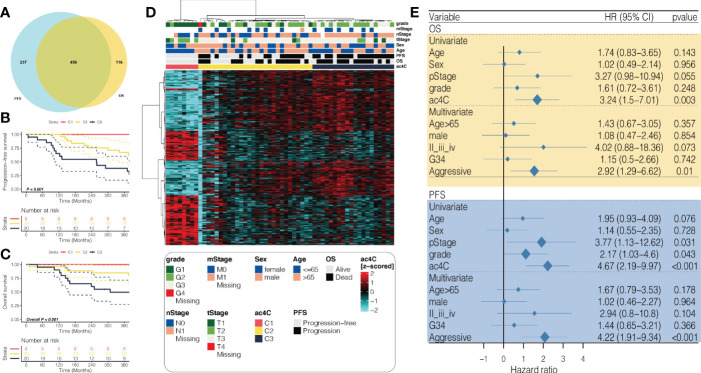
Identification of prognostic subtypes in PDAC based on NAT10-relevant gene set. **(A)** Venn diagram showing the intersection of prognostic NAT10-relevant genes regarding PFS in the left and OS in the right. **(B)** heatmap showing the unsupervised clustering using the prognostic NAT10-relevant genes in 56 PDAC cases in TCGA cohort. Kaplan-Meier curves for the three identified NAT10-relevant PDAC subtypes regarding both **(C)** PFS and **(D)** OS. **(E)** Forest plot showing the independent prognostic value of the NAT10-relevant subtypes after adjusting other clinical features.

To narrow down the gene list and search for the prognostication-relevant genes that were associated with NAT10, we conducted univariate analysis using Cox proportional hazard regression model in PDAC cases with sufficient tumor purity (n = 56), taking into account both PFS and OS. We identified 695 genes (**Table S1**) and 574 genes (**Table S2**) related to PFS and OS (*P* < 0.05), relatively, and a total of 458 genes were shared (**Figure 1A**). We then performed unsupervised clustering using these 458 NAT10-relegent prognostic genes and revealed three subtypes among PDAC cases in TCGA cohort (**Figure 1B**). The identified subtypes were tightly associated with PFS (*P* < 0.001; **Figure 1C**) and OS (*P* < 0.001; **Figure 1D**); notably, C3 showed the most inferior outcome as compared to other cases. We then investigated the prognostic value of our proposed NAT10-relavnt PDAC subtype, which we found remains an independent prognostic factor after adjusting for other clinical characteristics regarding PFS and OS (**Figure 1E**).

### 3.2 External validation of the prognostic subtypes in PDAC

**Figure 2:**
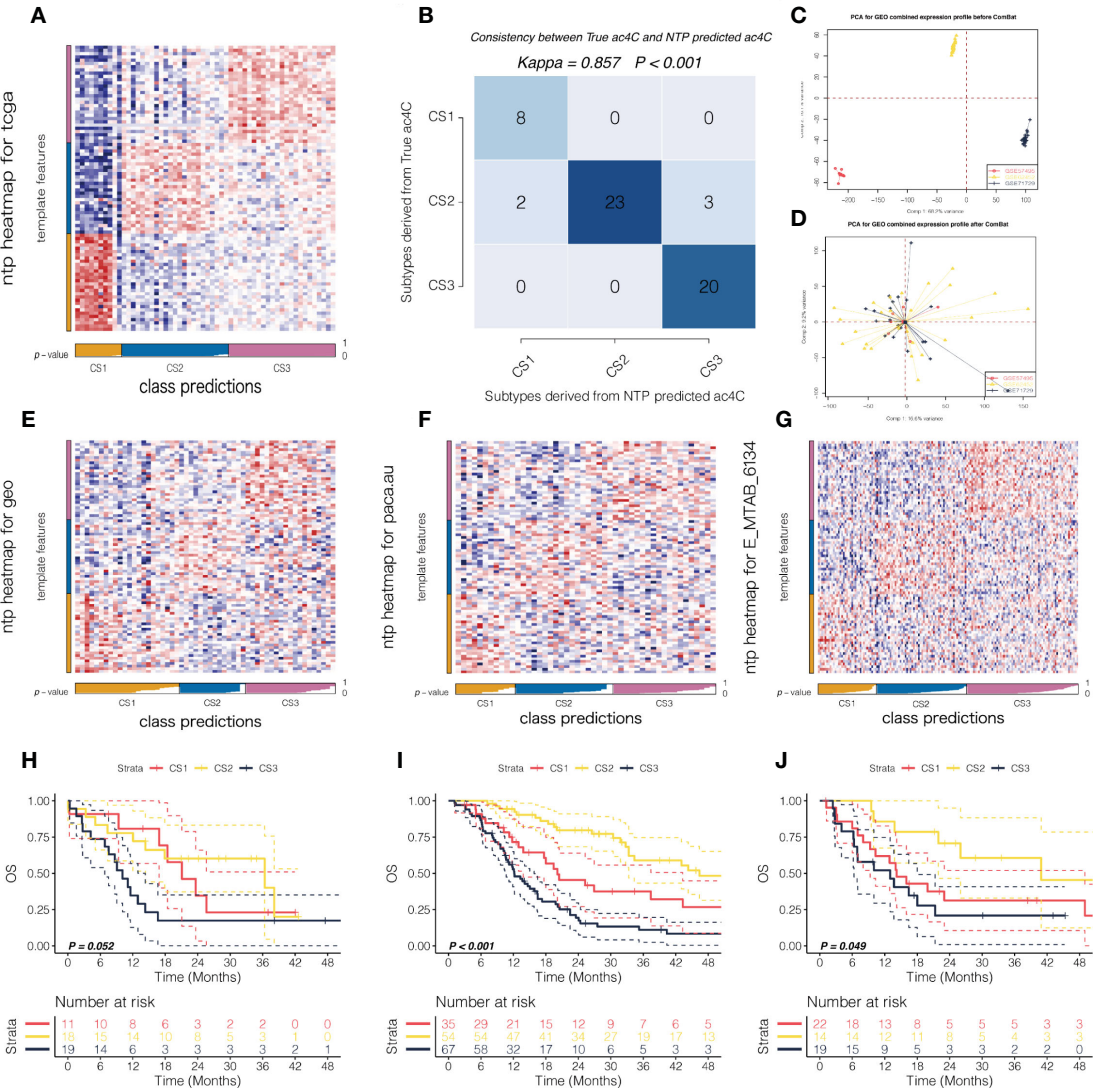
External validation of the prognostic subtypes in PDAC. **(A)** Heatmap showing the NTP results using 88-gene classifier in TCGA cohort. **(B)** Consistency map showing high agreement between true subtype and predicted subtype in TCGA cohort. PCA plot showing the potential batch effect **(C)** before and **(D)** after removal. NTP heatmap using 88-gene classifier was shown in **(E)** for GEO cohort, and the corresponding survival curves regarding OS were shown in **(F)**. Likewise, NTP heatmap using 88-gene classifier was shown in **(G)** for PACA_AU cohort and **(H)** for E-MTAB-6134 cohort, respectively. The corresponding survival curves regarding OS were shown in **(I)** for PACA_AU cohort and **(J)** for E-MTAB-6134 cohort, respectively.

In order to determine whether our newly identified NAT10-relevant prognostic subtypes can be reproducible in external cohorts of patients with PDAC, we tested their reproducibility. In this manner, we searched for the top 30 NAT10-relevant genes that were significantly and uniquely upregulated (log2FoldChange > 0, FDR< 0.05) in each of the three subtypes from TCGA cohort, ending up with a 88-gene classifier in which 30 genes for C1, 28 genes for C2, and 30 genes for C3 (**Table S3**). We then deployed the 88-gene classifier to the discovery TCGA cohort using NTP algorithm (**Figure 2A**); we demonstrated superior predictive accuracy with only five misclassified (Kappa = 0.857, *P* < 0.001; **Figure 2B**), which made us confident of using such classifier in external cohorts.

In this manner, we first deployed the 88-gene classifier to the combined GEO cohort with sufficient tumor purity (n = 55) where potential batch effect was removed and investigated by PCA (**Figure 2C, D**). NTP revealed three NAT10-relevant subtypes in GEO cohort (**Figure 2E**), which showed significantly separated OS rate and the predicted CS3 presented with the most unfavourable prognosis (**Figure 2F**). Using the same strategy, we reproduced three subtypes in PACA_AU (n = 48) and E-MTAB-6134 cohorts (n = 156), respectively (**Figures 2G, H**). Likewise, the reproduced subtypes demonstrated prognostication-relevance (*P* = 0.052 in PACA_AU cohort and *P* < 0.001 in E-MTAB-6134 cohort) and CS3 showed inferior outcome as compared to other subtypes (**Figures 2I, J**). C1, C2, C3 were the molecular typing of the training set, and CS1,CS2,CS3 are the molecular typing of the validation set.

### 3.3 Activation of immune- and cell cycle-related pathways in the aggressive subtype

**Figure 3:**
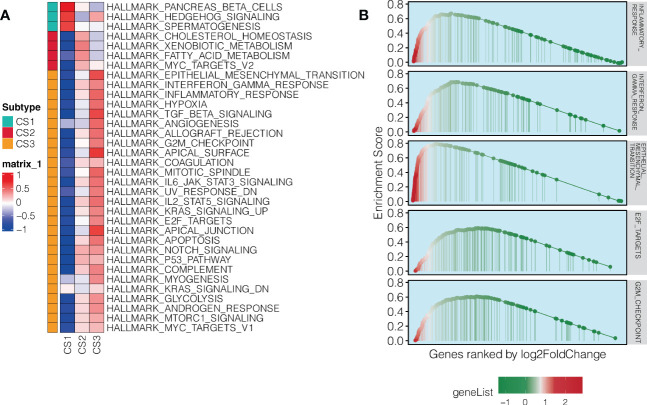
Pathway enrichment in the aggressive NAT10-relevant PDAC subtype. **(A)** Heatmap showing significantly and uniquely upregulated Hallmark pathways in each of the three NAT10-relevant PDAC subtypes; the input was the GSVA enrichment score of the specific pathway in specific subtype. **(B)** GSEA plot for immune-related and cell cycle-related pathways in the aggressive C3 subtype of PDAC in TCGA cohort.

To further understand the biology-relevance behind each of the three subtypes, we performed differential expression analysis and GSEA using Hallmark pathways. Significantly and uniquely upregulated Hallmark pathways (NES > 0, FDR < 0.05) were identified for each of the three subtypes,we found that the pathway represented by Pancreas beta cells was up-regulated in the CS1 subtype, and the pathway represented by MYC Targets V2 was up-regulated in the CS2 subtype. And the pathway represented by Epithelial mesenchymal translation is up-regulated in CS3 subtype. (**Figure 3A**; **Table S4**). Of note, we found immune-related pathways were significantly activated in the aggressive CS3 subtype as compared to other cases; these pathways included inflammatory response (NES: 1.86, FDR = 0.002), interferon-gamma response (NES: 1.90, FDR = 0.002), and epithelial mesenchymal transition (EMT; NES: 2.20, FDR = 0.002). In addition, cell cycle-relevant pathways (i.e., E2F targets [NES: 1.64, FDR = 0.002] and G2M checkpoint [NES: 1.68, FDR = 0.002]) were significantly upregulated in CS3 comparing to other subtypes (**Figure 3B**).

### 3.4 Differential tumor microenvironment landscape across PDAC

**Figure 4:**
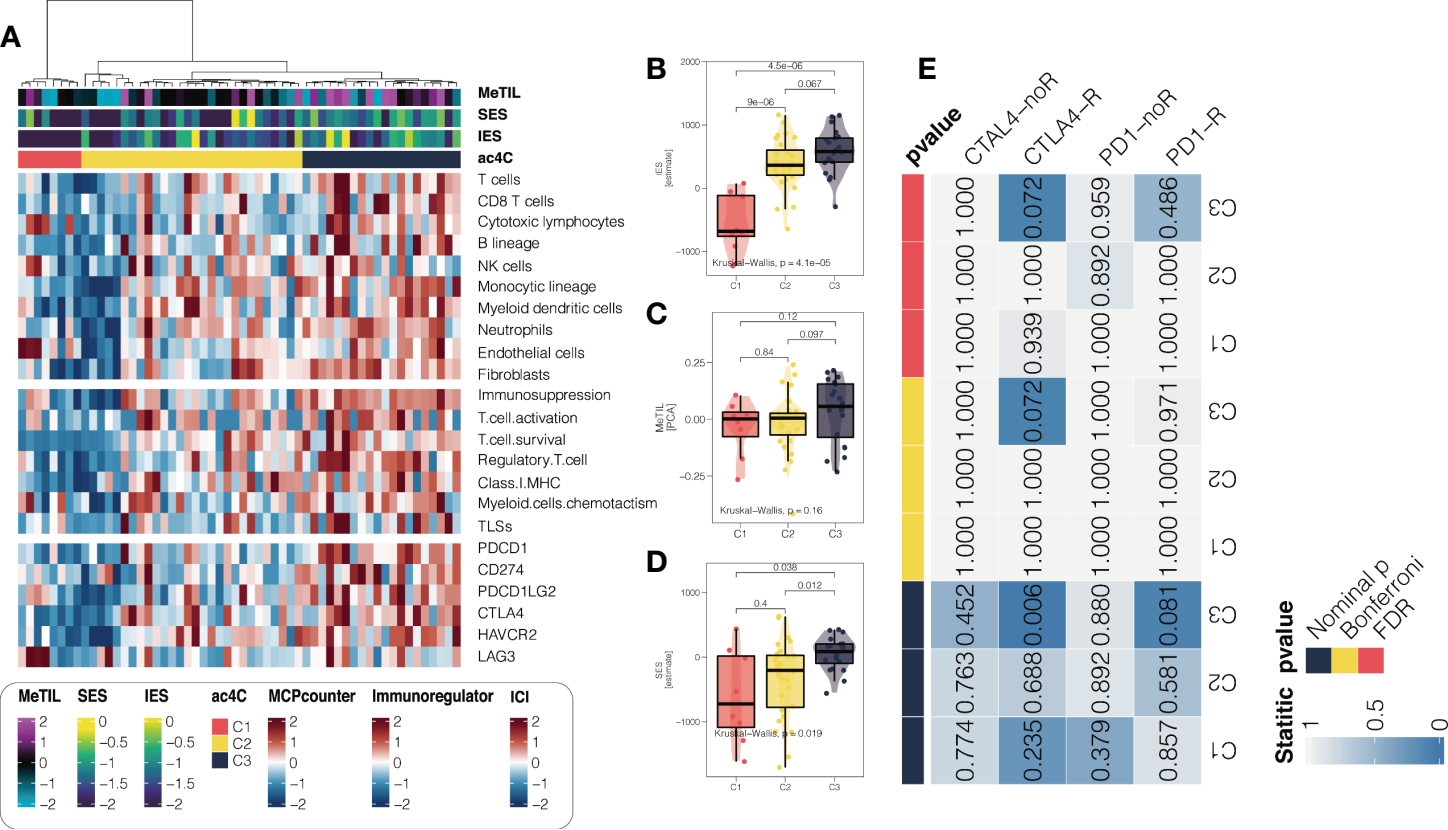
An analysis of the microenvironment of PDAC tumor subtypes associated with NAT10. **(A)** Heatmap showing the tumor microenvironment landscape in PDAC from the TCGA cohort, with the top panel showing the cell abundance estimated by MCPcounter algorithm, the middle panel showing the enrichment score of gene signatures for the functional orientation and the bottom panel showing expression of representative genes involved in immune checkpoint targets; the immune/stromal enrichment score and MeTIL score were annotated at the top of the heatmap. Distribution of immune enrichment scores, stromal enrichment scores and MeTIL scores among three NAT10-relevant subtypes in TCGA cohort were shown in **(B)**, **(C, D)**, respectively. **(E)** Subclass mapping demonstrating that the aggressive C3 subtype may be sensitive to immune checkpoint inhibitors.

As we have shown the immune dysfunction of among three subtypes, we then decided to in-depth profile the TME of PDAC. Therefore, we quantified global enrichment of immune/stromal cells and tumor-infiltrating lymphocyte based on DNA methylation data. We further investigated 10 TME cell infiltration levels in TCGA cohort, seven gene signatures for the functional orientation, and surveyed the PDAC samples for the expression of immune checkpoints (**Figure 4A**). We found that the aggressive C3 showed highly infiltrated with immune (**Figure 4B**), stromal cells (**Figure 4C**) and tumor-infiltrating lymphocytes (**Figure 4D**), and enriched in both immune activation and suppression factors. Moreover, C3 showed relatively higher expression of nearly all immunotherapy targets, including CD274, PDCD1, PDCD1LG2, CTLA4, and HAVCR2. As our findings demonstrated that C3 presented with highly infiltrated TME and activated interferon-gamma signaling pathway, we therefore hypothesized that C3 may have higher likelihood of responding to immune checkpoint inhibitor as compared to other subtypes. In order to accomplish this, we mapped subclasses in the TCGA cohort. According to the results, the transcriptome profiles of C3 and melanoma patients who responded to anti-CTLA4 immunotherapy were highly similar (*P* = 0.006, Bonferroni adjusted *P* = 0.072, FDR adjusted *P* = 0.072; **Figure 4E**). Our findings add to the literature that molecular classification using NAT10-relevant genes may be capable of identifying ideal candidates for immunotherapy in patients with PDAC.

### 3.5 Genetic delineation of the NAT10-relevant subtypes of PDAC

**Figure 5:**
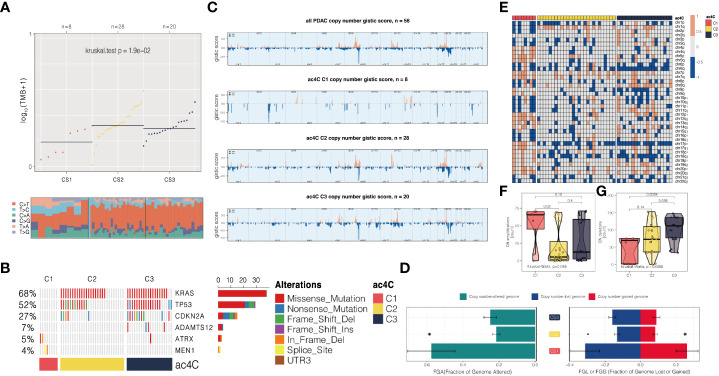
Three NAT10-relevant subtypes of PDAC in the TCGA cohort exhibit genomic heterogeneity and chromosomal instability. **(A)** An analysis of the distribution of TMB and TiTv (transition to transversion) among three subtypes is presented. **(B)** Onco printing showing the distribution of genes that were differentially mutated among three subtypes. **(C)** An overview of the CNA landscape across the entire cohort and among the NAT10-relevant subtypes. **(D)** An example of a barplot showing the distribution of FGA, FGG, and FGL. Bar charts are presented as means with standard errors of the means. **(E)** Heatmap showing arm-level CNA among three subtypes. Distribution of focal-level copy number amplifications and deletions across three subtypes were shown in **(F, G)**, respectively.

Furthermore, the genomic landscape plays an integral role in anti-tumor immunity as well as molecular features. Several factors can trigger T-cell responses, including tumor mutational burden (TMB) and the presence of neoantigens (38, 40), while aneuploidy may result in immune evasion and reduced response to immunotherapy (41). To the end, we first explored the genetic differences among three subtypes. We dissected the mutational landscape across all the samples and calculated the TMB for each tumors; we found that CS1 harboured significantly less TMB as compared to other cases (*P* = 0.019; **Figure 5A**). We then identified a total of six mutations that showed significantly differential rate of mutational frequency among three subtypes (*P* < 0.05); these mutations occurred in at least 3% of the PDAC in TCGA cohort (**Table S5**). We then investigated if these genes included any mutations that were previously identified as driver mutations for cutaneous melanoma (42), and three mutations were identified, including KRAS (68%), TP53 (52%), and CDKN2A (27%). Strikingly, none of these three mutations occurred in C1 (**Figure 5B**).

We then investigated chromosomal instability by first profiling the broad-level CNA across the whole human gene in the entire cohort, and three subtypes we proposed, respectively (**Figure 5C**). Consistently, we found that CS1 showed generally higher chromosomal instability as compared to other subtypes by calculating the individual FGA values as well as fraction genome gained and lost (FGG and FGL) (**Figure 5D**). Next, we profiled the focal-level CNA for PDAC (**Figure 5E**) and we found that C1 showed significantly higher focal-level CN amplifications (*P* = 0.065; **Figure 5F**); while C3 enriched in CN deletions (*P* = 0.009; **Figure 5G**) comparing to other two subtypes.

### 3.6 Association between DNA replication stress and NAT10-relevant subtypes

**Figure 6:**
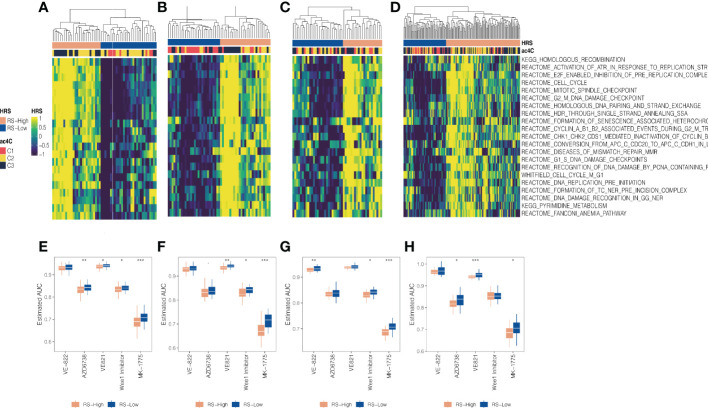
Association between DNA replication stress and NAT10-relevant subtypes in PDAC. Replication stress and DNA damage response activate pathways and molecular processes involved in DNA maintenance and cell cycle regulation. Two replication stress (RS) subtypes were identified for the **(A)** TCGA cohort, **(B)** GEO cohort, **(C)** PACA_AU cohort, and **(D)** E-MTAB-6134 cohort, respectively. **(D)** Boxplot showing distribution of estimated AUC concerning ATR and WEE1 inhibitors between two RS subtypes for the **(E)** TCGA cohort, **(F)** GEO cohort, **(G)** PACA_AU cohort, and **(H)** E-MTAB-6134 cohort, respectively. * means P < 0.05, ** means P < 0.01, *** means P < 0.001.

We investigated the potential of targeting RS (Replication stress) as a PDAC subgroup treatment regimen because we found that cell cycle-related oncogenic pathways are activated in aggressive C3 and activation of cell cycle pathways activates cell cycle checkpoint regulatory proteins involved in RS such as ATR and WEE1, which have been described as closely related proteins associated with DNA damage responses leading to cisplatin resistance (29). Unsupervised hierarchical clustering using 21 replication stress markers resulted in two RS isoforms (RS-High and RS-Low) in the TCGA, GEO, PACA AU and E-MTAB-6134 cohorts (**Figures 6A–D**). There was a significant enrichment of aggressive C3 in RS-High (all, P 0.05), indicating chemoresistance based on cisplatin. We estimated AUC for three ATR inhibitors (VE-821, VE-822, and AZD6738) and two WEE1 inhibitors (WEE1 inhibitor and MK-1775) provided by GDSC using a model-based prediction strategy similar to the literature (43). Cell cycle checkpoint inhibitors were generally more effective against the RS-High subtype in all four cohorts than against the RS-low (**Figures 6E–H**).

### 3.7 NAT10-relevant resistance to gemcitabine in PDAC

**Figure 7:**
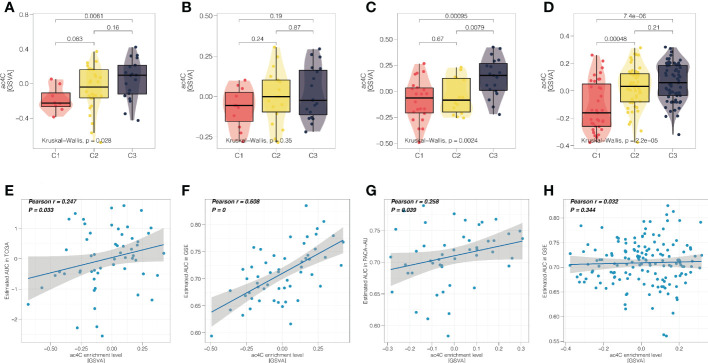
NAT10-relevant resistance to gemcitabine in PDAC. Distribution of single-sample enrichment score of NAT10 in three PDAC subtypes for the **(A)** TCGA cohort, **(B)** GEO cohort, **(C)** PACA_AU cohort, and **(D)** E-MTAB-6134 cohort, respectively. **(D)** Scatter correlation plot showing the Pearson’s correlation between the NAT10 enrichment score (x-axis) and predicted AUC of gemcitabine (y-axis) for the **(E)** TCGA cohort, **(F)** GEO cohort, **(G)** PACA_AU cohort, and **(H)** E-MTAB-6134 cohort, respectively.

Gemcitabine is the standard first-line treatment for unresectable locally advanced or metastatic pancreatic ductal adenocarcinoma since 1997 (44). In this context, we further investigated the association between NAT10-relevant subtypes and sensitivity of responding to gemcitabine. Firstly, we profiled the distribution of NAT10 in three PDAC subtypes, and we found that the single-sample level enrichment score of NAT10 tended to increase from C1 to C3 in four cohorts (**Figures 7A–D**). Secondly, we performed correlation analysis and we found that the predicted AUC of gemcitabine was positively correlated with the NAT10 enrichment score (**Figures 7E–H**), which indicated that PDAC patients with increasing NAT10 expression level may experience increasing resistance to treatment of gemcitabine.

### 3.8 NAT10 expression levels and prognosis in pancreatic ductal adenocarcinoma tissues analyzed online

**Figure 8:**
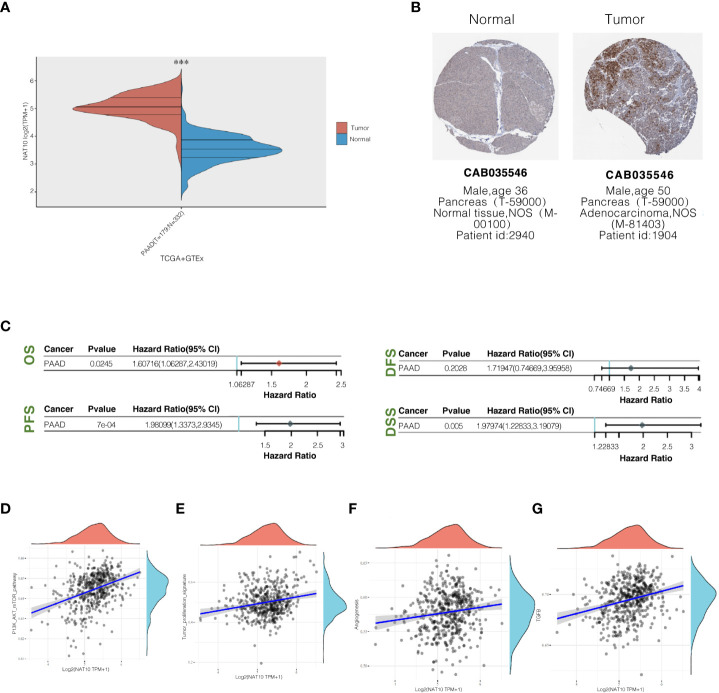
NAT10 expression levels in pancreatic ductal adenocarcinoma tissues and their relationship with prognosis. **(A)** Comparison of NAT10 expression levels in pancreatic ductal adenocarcinoma tissues and normal pancreatic tissues from TCGA and GTEx database sources; **(B)** The immunohistochemical staining of NAT10 in pancreatic ductal adenocarcinoma tissues and normal pancreatic tissues from the human protein atlas database. **(C)** Elevated NAT10 expression in the TCGA dataset predicts poor prognosis associated with patients. **(D, E)** Pathway correlation analysis of NAT10 positively correlates with the degree of PI3K-AKT activation and cell proliferation characteristics. **(F, G)** NAT10 positively correlates with TGF-β and angiogenic capacity.

Using the GEPIA website, NAT10 expression levels were analyzed between the TCGA and GTEx databases in pancreatic ductal adenocarcinoma tissues. Pancreatic ductal adenocarcinoma tissues expressed significantly higher levels of NAT10 than normal pancreatic tissues, and the difference was statistically significant (**Figure 8A**). A search from the Human Protein Atlas database showed that NAT10 expression was significantly higher in pancreatic ductal adenocarcinoma tissues (**Figure 8B**). An analysis of pancreatic ductal adenocarcinoma prognoses from the TCGA and GTEx databases can be found on the GEPIA website. According to the survival curve analysis, NAT10 expression levels were negatively correlated with OS, PFS, and DSS. Patients with abnormally high NAT10 expression had poor prognoses, with statistically significant differences (**Figure 8C**). AKT activation and cell proliferation characteristics were positively correlated (**Figures 8D, E**), suggesting that the abnormal expression of NAT10 may promote the malignant proliferation of pancreatic ductal adenocarcinoma by activating the PI3K-AKT pathway. On the other hand, NAT10 was positively correlated with TGF-β and angiogenic ability (**Figures 8F, G**).

### 3.9 NAT10 knockdown significantly inhibited the migration and clonogenic ability of pancreatic ductal adenocarcinoma cells and reduced the resistance to gemcitabine

**Figure 9:**
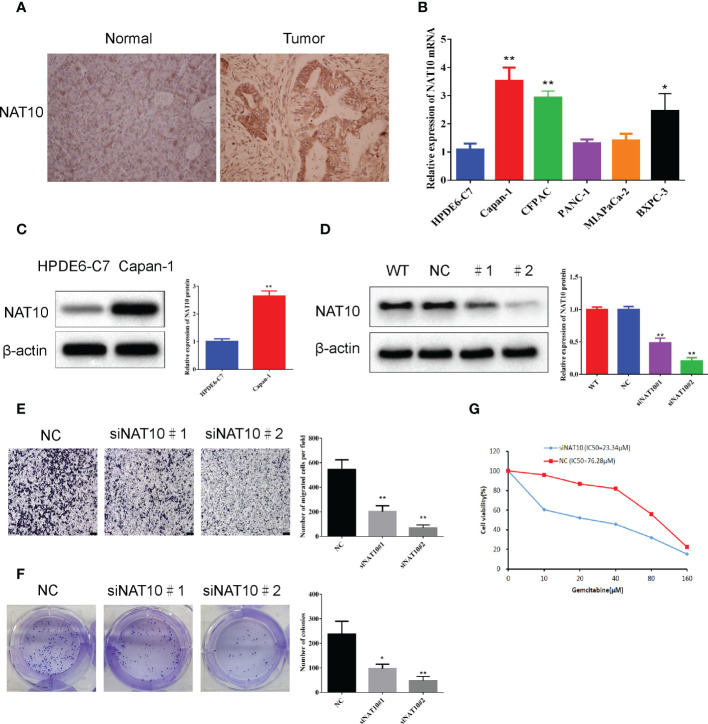
NAT10 knockdown significantly inhibited the migration and clonogenic ability of pancreatic ductal adenocarcinoma cells. **(A)** Expression of NAT10 in tumor and normal tissues. **(B)** The qRT-qPCR analysis showed that the expression level of NAT10 was significantly higher in the PDAC cell lines compared to normal pancreatic ductal epithelial cells. **(C)** The Western blot analysis showed that the expression level of NAT10 was significantly higher in the Capan-1cell line compared to normal pancreatic ductal epithelial cells. **(D)** NAT10 expression in Capan-1cells after NAT10-specific siRNA knockdown. **(E)** Significant reduction in migrating cells in the siRNA group relative to negative control following NAT10-specific siRNA knockdown. **(F)** The reduced clonogenic capacity of Capan-1cell line after specific siRNA knockdown of NAT10. **(G)** The IC50 of gemcitabine in Capan-1cells after specific siRNA knockdown of NAT10. The data are the mean ± SEM. *P<0.05, **P<0.01.

The expression of the NAT10 gene in in tumor and normal tissues, and the results are shown in (**Figure 9A**). There was a significant increase in NAT10 expression in the Capan-1 cell line in comparison to normal pancreatic ductal epithelial cells (P<0.05) (**Figures 9B, C**). The role of NAT10 in tumorigenicity was studied more thoroughly by transfecting Capan-1cells with a specific siRNA targeting it. We designed two siRNAs to knock down NAT10. Western blot analysis confirmed the effectiveness of NAT10 knockdown, and both siRNAs significantly knocked down NAT10, and the protein level of NAT10 were barely detectable after transfection with siRNA#2 (**Figure 9D**). The data demonstrate that the siRNA targeting NAT10 has high specificity and transfection efficiency. In both siRNA-transfected cells, cell proliferation was inhibited with increasing time. According to the Capan-1 cell line colony formation assay, knockdown of NAT10 led to a reduction in colonies (**Figure 9E**). By knocking down NAT10, pancreatic ductal adenocarcinoma cells are inhibited from proliferating and forming colonies. Transwell analysis also revealed that specific siRNA inhibited migration of cells knockdown of NAT10 (**Figure 9F**). In addition, the siRNA targeting NAT10 reduced the IC50 of gemcitabine in Capan-1 cells from 76.28μM to 23.34μM (**Figure 9G**), indicating that NAT10 knockdown significantly reduced the resistance of pancreatic ductal adenocarcinoma cells to gemcitabine.

## 4 Discussion

PDAC is an essential and growing global health problem. The overall treatment of pancreatic ductal adenocarcinoma is poor (45), and the synergistic treatment of cancer cells and mesenchymal targeting, reversal of inhibitory immune responses, and antitumor activity is now probably the most promising approaches of PDAC (46). In other words, combining chemotherapy, immunotherapy, targeted therapy, and stromal-targeted drugs is the most promising direction for pancreatic ductal adenocarcinoma treatment research (10). Today’s medical era is one of precision, and the guidance of biomarkers can lead to optimal therapeutic management patterns. Increasing numbers of well-performing cancer prognostic models have been developed by researchers for the detection and prognostic evaluation of cancer (47–49). However, their clinical application still needs to be validated by numerous trials to investigate the most appropriate procedures to identify and follow the status of therapeutic management patterns.

NAT10 is a nucleoprotein involved in histone acetylation and has the potential for subcellular redistribution. NAT10 activates rRNA transcription by acetylating upstream binding factors and recruiting RNA polymerase I-related factor 53 and RNA polymerase I to rDNA (50). Based on the NAT10-related gene set, we identified prognostic subtypes of the PDAC dataset in the TCGA database. Cox proportional hazards regression models performed univariate analysis on PDAC cases with sufficient tumor purity. A total of 458 co-expressed genes were identified. Unsupervised cluster analysis revealed three subtypes of PDAC cases in the TCGA cohort, and the subtypes were strongly associated with PFS and OS, with the C3 subtype showing the worst outcome. We then used the GEO database PDAC for reproducibility testing of NAT10-related prognostic subtypes. The results showed excellent predictive accuracy. We further performed differential expression analysis and GSEA using the Hallmark pathway to understand the biological relevance behind the three subtypes. Immune-related pathways such as inflammatory response, interferon-gamma response, and epithelial-mesenchymal transition were significantly activated in the aggressive C3 subtype compared to other cases; cell cycle-related pathways were also significantly upregulated in C3, indicating that immune activation was associated with prognosis of pancreatic ductal adenocarcinoma was negatively correlated. We further investigated the level of infiltration of 10 TME cells in the TCGA cohort, the functional localization of 7 gene signatures, and the expression of immune checkpoints in PDAC samples. The findings suggest that C3 presents highly infiltrated TME and activated interferon-γ signaling pathways. We, therefore, hypothesize that C3 may have a higher response to immune checkpoint inhibitors compared to other subtypes’ likelihood.

For this reason, molecular classification using NAT10-related genes may be able to identify ideal candidates for immunotherapy in PDAC patients. In addition to molecular features, genomic status is also inextricably linked to antitumor immunity; we explored genetic differences between the three subtypes. We found that C1 had significantly fewer TMBs. We identified six mutations that showed significant differences in mutation frequency across the three subtypes; we investigated chromosomal instability by first profiling CNA at the broad level across the entire human gene in the cohort, as well as each of the three subtypes we proposed. We found that CS1 generally exhibited higher chromosomal instability compared to the other subtypes. Analysis of focal-level CNA of PDAC showed that C1 had significantly higher amplification of focal-level CN, while C3 had abundant deletions of CN compared to the other two subtypes.

As a result, we explored whether targeting RS could be a therapeutic option for PDAC patients. RS-high was significantly enriched in aggressive C3, suggesting potential cisplatin-based chemoresistance. We further investigated the relationship between NAT10-related subtypes and sensitivity to gemcitabine response. We found a trend of increasing single-sample level enrichment fraction of NAT10 from C1 to C3 across the four cohorts. Secondly, the predicted AUC of gemcitabine was positively correlated with the enrichment fraction of NAT10, suggesting that PDAC patients with increasing levels of NAT10 expression may be increasingly resistant to treatment with gemcitabine.

NAT10 expression levels in pancreatic ductal adenocarcinoma tissues from the TCGA and GTEx databases showed that NAT10 expression levels were significantly higher in pancreatic ductal adenocarcinoma tissues than in normal pancreatic tissues. The results of the survival curve analysis showed that NAT10 expression levels were negatively correlated with OS, PFS, and DSS and that abnormally elevated expression was predictive of poor prognosis in patients. That pathway correlation analysis revealed that NAT10 was positively correlated with PI3K-AKT activation and cell proliferation characteristics, suggesting that abnormal expression of NAT10 may promote the malignant proliferation of pancreatic ductal adenocarcinoma through activation of the PI3K-AKT pathway. To further clarify whether the above correlation analysis was valid, we first analyzed the expression of NAT10 in tumor and normal tissues, selected Capan-1for further study, and found that the expression level of NAT10 was significantly higher in pancreatic ductal adenocarcinoma cell line Capan-1compared to normal pancreatic ductal epithelial cells. Based on this, we used two different siRNAs to knock down NAT10 and verified the knockdown efficiency of the siRNAs by WB. We evaluated the differential changes in cell migration and clonogenic ability after the NAT10 knockdown based on the above correlation analysis. We found that: NAT10 knockdown significantly inhibited the migration and clonogenic ability of pancreatic ductal adenocarcinoma cells. In addition, as to how NAT10 plays a role in promoting cancer, the following arguments are selected to support the above experimental results: NAT10 promotes tumor cell migration and EMT transformation by regulating mRNA N4-acetylcytidine (ac4C) modification pathway. And NAT10, by offering the ubiquitin USP39, prevents its ubiquitination dependent degradation. As the key regulatory factor of mRNA ac4c modification, the NAT10 is shown to maintain the stability of the oncogene expression and improve its translation efficiency through ac4c modification (51, 52).

## 5 Conclusion

To conclude, this study examined the correlation between NAT10 expression levels and pancreatic ductal adenocarcinoma patients’ clinical characteristics. We constructed a prognostic prediction model for pancreatic ductal adenocarcinoma based on genes involved in NAT10-mediated ac4C modification using data related to pancreatic ductal adenocarcinoma patients from the TCGA, GEO databases for the diagnosis and treatment of pancreatic ductal adenocarcinoma.

## Data availability statement

The original contributions presented in the study are included in the article/**Supplementary Material**. Further inquiries can be directed to the corresponding authors.

## Author contributions

HY and CX designed the study. DX, KH, YC, and FY performed data analysis. DX and KH drafted the manuscript. HY and CX revised the manuscript. All authors read and approved the final manuscript.
